# Peer Review of “Effects of Interventions for the Prevention and Management of Maternal Anemia in the Advent of the COVID-19 Pandemic: Systematic Review and Meta-Analysis”

**DOI:** 10.2196/81700

**Published:** 2025-10-06

**Authors:** I Gde Sastra Winata

**Affiliations:** 1 Udayana University, Bali, Indonesia

**Keywords:** maternal anemia, anemia in pregnancy, COVID-19, pregnancy complications, meta-analysis, maternal and child health, anemia prevention, reproductive health

*This is the peer-review report for “Effects of Interventions for the Prevention and Management of Maternal Anemia in the Advent of the COVID-19 Pandemic: Systematic Review and Meta-Analysis.”*

## Round 1 Review

### General Comments

This paper [1] addresses a relevant and important topic in maternal health, particularly in the context of anemia during pregnancy. The paper has potential for publication in a journal with minor revisions.

### Specific Comments

#### Major Comments

1. There is no conclusion in this manuscript. Add a Conclusion section that summarizes the content of this study.

#### Minor Comments

2. Introduction section. Briefly explain the types of interventions implemented during COVID-19 to prevent anemia in pregnant women. Provide a brief explanation of the differences in anemia prevention interventions before and after COVID-19.

3. Discussion, at the end of the Discussion section. Include the main findings of this study and emphasize their significance in addressing anemia-related challenges. Highlight the contribution of the study results to public health, particularly how the findings can inform or improve anemia prevention and treatment strategies in health care systems. Provide practical recommendations or actionable steps based on the study’s outcomes that can be implemented in maternal health care policies and programs.
